# Bio-electrospraying and aerodynamically assisted bio-jetting the model eukaryotic *Dictyostelium discoideum*: assessing stress and developmental competency post treatment

**DOI:** 10.1098/rsif.2010.0696

**Published:** 2011-02-02

**Authors:** Nicholl K. Pakes, Suwan N. Jayasinghe, Robin S. B. Williams

**Affiliations:** 1 Centre for Biomedical Sciences, School of Biological Sciences, Royal Holloway University of London, Egham, Surrey TW20 0EX, UK; 2 BioPhysics Group, Department of Mechanical Engineering, University College London, Torrington Place, London WC1E 7JE, UK

**Keywords:** aerodynamically assisted bio-jetting, bio-electrosprays, *Dictyostelium*, *gapA*, stress response, *yakA*

## Abstract

Bio-electrospraying (BES) and aerodynamically assisted bio-jetting (AABJ) have recently been established as important novel biospray technologies for directly manipulating living cells. To elucidate their potential in medical and clinical sciences, these bio-aerosol techniques have been subjected to increasingly rigorous investigations. In parallel to these studies, we wish to introduce these unique biotechnologies for use in the basic biological sciences, for handling a wide range of cell types and systems, thus increasing the range and the scope of these techniques for modern research. Here, the authors present the analysis of the new use of these biospray techniques for the direct handling of the simple eukaryotic biomedical model organism *Dictyostelium discoideum*. These cells are widely used as a model for immune cell chemotaxis and as a simple model for development. We demonstrate that AABJ of these cells did not cause cell stress, as defined by the stress-gene induction, nor affect cell development. Furthermore, although BES induced the increased expression of one stress-related gene (*gapA*), this was not a generalized stress response nor did it affect cell development. These data suggest that these biospray techniques can be used to directly manipulate single cells of this biomedical model without inducing a generalized stress response or perturbing later development.

## 1. Introduction

The ability to directly handle living biological materials such as biological cells and whole organisms for precise placement (in the *x*, *y* and *z*-axes) suggests a huge spectrum of functionality, ranging from basic biological research to wide spread applications in the clinic [1,2]. In particular, the development of such technology could have far-reaching implications for tissue engineering and regenerative biology and medicine. In the race to develop this technology, non-contact jet-based methodologies lead the field as they have the capability to directly handle and place living cells and organisms within a polymer matrix in true three dimensions [3]. These technologies will thus enable the formation of three-dimensional fully functional cell cultures in a wide range of biological systems, with the ultimate goal of allowing three-dimensional human cell culture research.

Bio-electrospraying (BES) explores a potential difference between two electrodes for generating a mist of charged cell-bearing droplets [4]. Contrary to BES, aerodynamically assisted bio-jetting (AABJ) is a process where a cell-bearing mist of droplets is generated by means of an applied pressure over an exit orifice [5]. Both BES and AABJ have clearly demonstrated efficacy in handling a wide range of living materials spanning from immortalized, primary and stem cells to those of whole organisms without compromising their viability and function [5–11]. These previous studies have spanned a wide range of investigations from genetics, genomics to physiological level studies, and these have established BES and AABJ as the leading technologies in this field [4,12–19]. Another such non-contact jet-based technology, ink-jet printing has also been investigated for handling living cells. This technology to date has not undergone a full in-depth investigation, in a biological standpoint, where the post-treated cells have been compared with controls as populations, where studies assess cells at a genetic, genomic to physiological level, thus, leaving this technology with several unanswered questions [2]. Therefore, these studies increase the importance of BES and AABJ as technologies to handle cells without damage.

To test the effect of these biospray techniques, we have chosen a well-established unicellular model eukaryotic organism, *Dictyostelium discoideum*. This eukaryotic social amoeba has an unusual life cycle whereby it reproduces by binary fission until local food sources are exhausted [20]. Starvation then initiates the developmental part of its life cycle, whereby cells aggregate and develop to form fruiting bodies of approximately 1 mm in height. The fruiting bodies consist of differentiated stalk and spore cells and the resulting spore cells are resistant to adverse environmental factors. Since signalling, movement and differentiation play major roles in the *Dictyostelium* life cycle, these processes have been widely investigated to better understand the fundamental mechanisms involved [21,22]. In these experiments, *Dictyostelium* is often used as a simple model to analyse human cell movement [23–25]. New roles for *Dictyostelium* have also been developed as a basic biomedical model [26], in, for example, the investigation of neuropsychiatric drug targets [27–30], in Alzheimer's disease research [31], in investigating the mechanisms of microbial infection [32] and in the analysis of the cellular role of defined proteins identified within the genome [33].

Therefore, here, we investigate the use of BES and AABJ in directly handling *Dictyostelium* cells. Cells processed with these biotechniques are assessed using two approaches: stress response, as identified by increased transcription of stress-related genes, and alterations in developmental patterning, as identified by the fruiting body formation and morphology. Our data indicate that AABJ had no effect on stress induction and did not cause an alteration in development, and BES caused an induction of one stress-related gene (out of three) post treatment and also did not cause an alteration in development. These data confirm the legitimacy of these biospray techniques in handling a wide range of cells.

## 2. Material and methods

### 2.1. Bio-aerosol techniques for cell handling

The BES and AABJ devices used in these studies are similar to those explored in our previous investigations ([1,6,7,10,11,17,18]: see the electronic supplementary material for equipment set-ups). Briefly, the BES and AABJ devices were both single needle units. In the case of BES, the needle had an inner bore diameter of approximately 800 µm and had a wall thickness of approximately 200 µm. The electrodes were separated over a distance of approximately 15 mm and a voltage of up to 15 kV was applied. In the case of the AABJ, the needle had an inner bore diameter of approximately 900 µm and a wall thickness of approximately 400 µm. For AABJ, we employed an applied pressure of approximately 0.5 bar within the chamber. Both these techniques were optimized using cell suspensions over a large parametric window of operation, which ranged from 1 to 30 kV for BES and 0.01–1 bar for AABJ. During these studies, high-speed photography was used for fully establishing the best parameters for a given technique, which would enable complete collection of the jetted suspension.

### 2.2. *Dictyostelium* cell culture

Wild-type *Dictyostelium* (Ax2) cells were cultured at 22°C in Axenic medium (ForMedium Ltd) containing 100 µg ml^−1^ penicillin and 100 µg ml^−1^ streptomycin. Prior to all experiments, cells were washed twice in phosphate buffer (3.8 mM K_2_HPO_4_, 16.5 mM KH_2_PO_4_), re-suspended at 1 × 10^7^ cells ml^−1^ in phosphate buffer and shaken in suspension at 120 r.p.m. for 4 h prior to biospraying or stress induction. Control stress-induction experiments comprised osmotic, heat and oxidative shock. For osmotic stress induction, cells were incubated with 200 mM sorbitol and shaken for 30 min to allow gene induction, as previously described [34]. For heat and oxidative stress induction, cells were either placed in a 33°C heat block for 30 min or combined with 50 µM dinitrophenol (DNP) and shaken for 30 min, respectively, as previously described [34]. To assess stress induction under osmotic, heat, oxidative or post-treated conditions, RNA was extracted from the cells using the High Pure RNA Isolation kit (Roche Ltd) and contaminating DNA was removed using a DNase*free* kit (Ambion, Inc.). cDNA was synthesized from 1 µg of RNA for each sample using the First Strand cDNA synthesis kit with Oligo(dt)18 primers (Fermentas Ltd). cDNA was amplified by PCR reaction using 10 pmol of the following primers; *yakA* (AATCGTTGAGATGCGTGG, GTGTACTAAATAGTTGAAGTGGTTG) *gapA* (TGGCCAAGTAGTGTGGTTCA, GTGTGTCGGTGAAATTCAATCCC), *rtoA* (TGGATCCTCATCTGATGGTAAA, AATCGTTGAGATGCGTGG), *glcS* (GTGGTATCTATCATCGTCATTGG, TTCACCCATCGCCTCG). Primers were designed, where possible, to flank introns (*gapA*, *yakA* and *glcS*), thus confirming cDNA amplification owing to the decrease in size of the cDNA-derived product (in comparison with the genomically derived product). The expression control product, glycogen synthase (GlcS; [35]), has been shown to be constantly expressed over development. Gene expression was semi-quantitatively assessed following agarose gel electrophoresis and using Quantity One software (Bio-Rad Ltd). Samples were analysed from at least triplicate independent experiments.

### 2.3. *Dictyostelium* development assay

To assess the effect of BES or AABJ on *Dictyostelium* cell development, assays were employed as previously described [36]. Briefly, 1 × 10^7^ cells subject to each spraying technique (and non-sprayed controls) were washed twice with phosphate buffer and transferred to nitrocellulose filters (Millipore Ltd). Development was induced at 22°C over a 24 h period and images were captured using QImaging RetigaE Fast1394 digital camera and Qcapture Pro software.

## 3. Results

**Figure 1.**
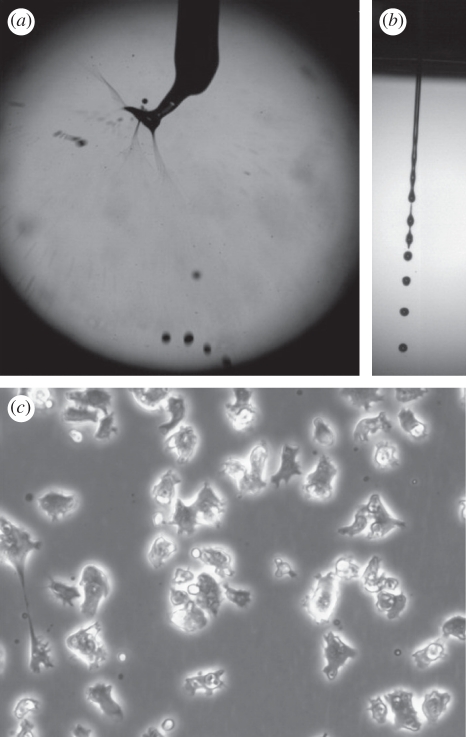
Biospraying of *Dictyostelium* cells. Representative high-speed images of (*a*) the bio-electrospraying (BES) and (*b*) the aerodynamically assisted bio-jetting (AABJ) of the *Dictyostelium* cells. (*c*) A characteristic optical micrograph of the cells, which were phenotypically indistinguishable between the controls and the post-treated cells.

Monitoring the physical properties of the biospray techniques employed here for *Dictyostelium* shows that BES takes place in an unstable jet (figure 1*a*), as a result of the properties (e.g. electrical conductivity and viscosity) of the cell suspension. This effect has also been observed for other cell-type suspensions [1,6–11,17]. In contrast, AABJ gives rise to a stable and continuous jet as this process employs a constant pressure (figure 1*b*). No gross physical differences were distinguishable at a cell physiological level following treatment with either jetting protocol in comparison with cells without spraying (figure 1*c*).

**Figure 2.**
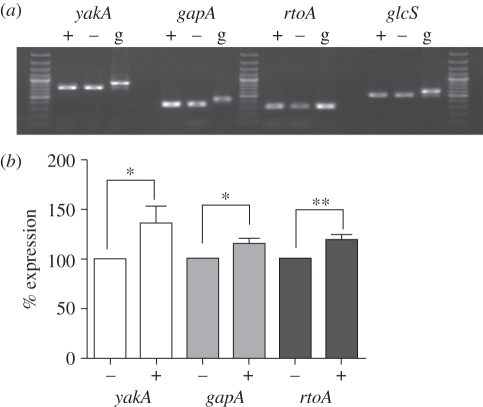
Expression of *Dictyostelium* stress-related genes following osmotic stress. (*a*) Monitoring gene expression by reverse-transcriptase PCR for three stress-related genes; *yakA*, *gapA* and *rtoA*, and a control (*glcS*) in presence (+) or absence (−) of 200 mM sorbitol. A genomic DNA control (g) for each gene is also provided. Three lanes of DNA size markers are included. (*b*) Results are shown as % expression compared with the control (100%) where *n* ≥ 3 independent experiments ±s.e.m. *p < 0.05, **p < 0.01.

**Figure 3.**
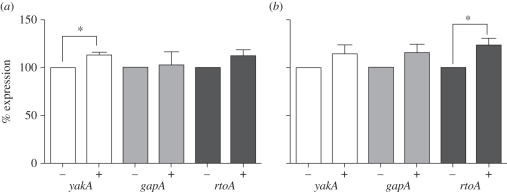
Expression of *Dictyostelium* stress-related genes following (*a*) oxidative and (*b*) heat stress. (*a*) Monitoring gene expression by reverse-transcriptase PCR for three stress-related genes; *yakA*, *gapA* and *rtoA*, and a control (*glcS*) in presence (+) or absence (−) of (*a*) 50 µM dinitrophenol (DNP—Oxidative) and (*b*) heat (33°C). (*b*) Results are shown as % expression compared with the control (100%) where *n* ≥ 3 independent experiments ±s.e.m. *p < 0.05.

Prior to assessing the effect of these biospray techniques on *Dictyostelium*, we first confirmed the stress-induced transcription response of three genes previously reported to increase transcription levels following stress induction: *yakA* [37], *gapA* and *rtoA* [34] (figure 2*a*). We chose a housekeeping gene, *glcS* [35] as a control for these experiments. Osmotically triggered stress, previously shown to induce the expression of *yakA*, *gapA* and *rtoA* genes, caused a highly significant increase in *rtoA* expression (19%; *p* < 0.01) and a significant increase in *gapA* and *yakA* expression (16 and 36%, respectively; *p* < 0.05, figure 2*b*) compared with control under test conditions. In comparison, treatment of cells with either oxidative or heat stress, as previously published [34,37], gave rise to specific increases in gene transcription (figure 3). Oxidative stress treatment with DNP (50 µM) caused a 13 per cent increase in *yakA* transcription (*p* < 0.05, figure 3*a*), whereas heat-induced stress (33°C, 30 min) caused a 23 per cent increase in *rtoA* transcription only (*p* < 0.05, figure 3*b*).

**Figure 4.**
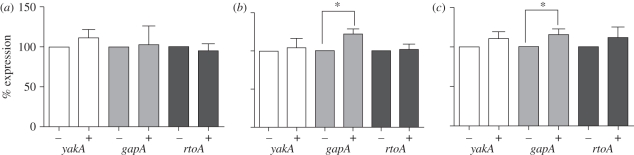
Effect of the biospraying processes on *Dictyostelium* stress response. Cells were sprayed using (*a*) AABJ with an applied pressure of 0.5 bar, and (*b*,*c*) using BES with an applied voltage of 10 and 15 kV, respectively. Stress response was monitored by reverse-transcriptase PCR for *yakA*, *gapA* or *rtoA*. Results are shown as % expression compared with the control (100%) where *n* ≥ 3 independent experiments ±s.e.m. *p < 0.05.

To assess the effect of these biospray techniques on *Dictyostelium*, cell suspensions were sprayed using AABJ and BES, and cells were allowed to recover for 30 min prior to RNA extraction for stress-induced transcriptional analysis. In these experiments, a baseline control was prepared without the jetting treatment and all samples were standardized using a transcription control (*glcS*). Analysis of the expression of each stress-related gene (*yakA*, *gapA* and *rtoA*) following biospray treatment showed no alteration in transcription for any of the genes following AABJ, indicating a lack of stress response caused by this handling technique (figure 4*a*). For BES, under two applied voltages, no significant increase in gene expression was seen for *yakA* and *rtoA*, although *gapA* showed a significant increase for both 10 and 15 kV BES conditions (15 and 22%, respectively, compared with control, *p* < 0.05; figure 4*b*,*c*).

**Figure 5.**
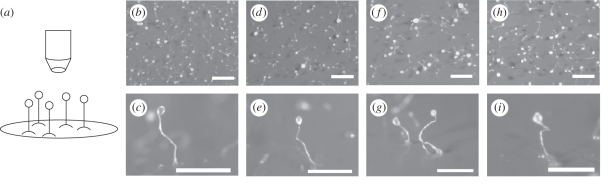
Effect of the biospraying processes on *Dictyostelium* development. Cells were sprayed and plated to enable development over a 24 h period prior to recording fruiting body morphology. (*a*) Schematic diagram illustrating fruiting body morphology. Development into mature fruiting bodies is shown for cells from (*b,d,f,h*) top-down (*c,e,g,i*) and side-angle views, respectively, for cells (*b*,*c*) without biospraying (controls), for cells following (*d*,*e*) AABJ at an applied pressure of 0.5 bar, and (*f*,*g*,*h*,*i*) BES at an applied voltage of 10 and 15 kV, respectively. Scale bar, (*b*,*d*,*f*,*h*), 1 mm; (*c*,*e*,*g*,*i*) 0.5 mm.

The induction of transcriptionally regulated stress response represents a transient, acute response to environmental stress. However, this does not reflect long-term changes in cell physiological integrity or behaviour following stressful handling. To examine this, we monitored changes in development for *Dictyostelium* post-biospray. Cells left to recover on nitrocellulose filters following AABJ or BES showed wild-type development and fruiting body morphology (figure 5). This is seen both in the population of fruiting bodies and in the shape and size of fruiting bodies post spraying.

## 4. Discussion and conclusions

The two biospray systems examined in this paper using *Dictyostelium* as a model, AABJ and BES, gave rise to distinct jet behaviour, as has been observed by us elsewhere (figure 1*a*,*b*) [1,6–11,17]. These differences are a consequence of the respective handling systems, whereby both systems employ different driving mechanisms for generating cell-bearing droplets. Although neither method indicated any basic cell damage at a visible level (figure 1*c*), we further monitored the effect of both handling techniques in the induction of cellular stress response.

No studies have previously looked at the physical stress induction of stress response gene expression in *Dictyostelium*. The most examined stress response pathway in this model, that of hyperosmotic stress, has suggested a signalling pathway involving tyrosine phosphorylation and subsequent actin cytoskeletal organization [38–41]. This pathway has also been suggested to function in a range of other stress-related stimuli including heat shock, and heavy metal exposure. For hyperosmotic shock, however, two other potential pathways have also been implicated in cellular stress responses: cAMP and DokA [42,43]; and guanylyl cyclase, cGMP and myosin II heavy chain [44–46]. Therefore in *Dictyostelium*, it is unclear if a common signalling pathway is responsible for all stress responses, thus we chose a range of stress-related genes to analyse for induction owing to physical stress.

To examine stress-related signalling, a study by Araki *et al.* [34] employed a microarray procedure to identify *gapA* and *rtoA* as genes that were induced under hyperosmotic stress. It is interesting to note that both these genes are induced by Dd-STATc in the JAK/STAT signalling pathway common to metazoa, since hyperosmotic induction of gene transcription was lost in the Dd-STATc null mutant. These authors also reported a stress-induced response following heat or oxidative stress, through Dd-STATc tyrosine phosphorylation, although an increase in gene expression was not examined. Another study by Taminato *et al.* [37] suggested that the kinase YakA functions in a PKA-dependent pathway in the response to oxidative and heat stress, and knockout mutants for *yakA* are hypersensitive to these stress conditions. Our data confirm *gapA*, *rtoA* and *yakA* are commonly induced under hyperosmotic shock (figure 2) in comparison with the loading control *glcS*. In contrast, our results show specificity for gene expression based upon other mechanism of stress induction, whereby heat showed a significant increase in *rtoA* expression and oxidative stress showed an increase in *yakA* expression (figure 3). These results therefore support a common stress-induction pathway via hyperosmotic stress induction, in addition to a mechanism-specific dependence for gene expression with oxidative and heat stress.

To analyse potential stress induction through biospraying, we found that AABJ has no significant effect on the expression of any of the three stress-related genes examined here. By contrast, BES caused a significant induction of expression of *gapA*, but had no effect on *rtoA* of *yakA* expression levels (figure 4). This suggests a mechanism-specific stress induction through BES, giving rise to changes in transcriptional regulation of specific genes. Further research into *Dictyostelium* stress responses and handling conditions will establish the specificity of this response.

Although transient stress induction provides an indication of immediate cellular stress, the experiments do not illustrate chronic changes in cell behaviour. Since *Dictyostelium* is a widely used model system for understanding immune cells chemotaxis [23–25], we therefore tested the effect of biospraying on *Dictyostelium* development (figure 5). Both AABJ and BES did not affect *Dictyostelium* development, a process critically involving directional cell movement (chemotaxis), and this suggests that these direct cell handling techniques may have a little effect on analogous mammalian systems, such as leucocytes. An interesting point the reader should note is that, BES explores high voltages but very low currents (in the nano-amperes), thus not compromising the cells membrane integrity as in those electrophoresis studies [47]. In addition, both these techniques unlike in the case of ink-jet printing apply the physical forces or driving mechanisms on the cells indirectly, which significantly reduces the triggering of cellular death (early or late apoptosis) as seen from these and our previous investigations. These techniques having been demonstrated as being inert to the direct handling of a wide range of cells and whole organisms can now undergo exploration for a wide range of applications spanning, a novel route for tissue engineering to the development of three-dimensional cultures most useful for modelling human diseases.
